# Expression of 3-Mercaptopyruvate Sulfurtransferase in the Mouse

**DOI:** 10.3390/molecules21121707

**Published:** 2016-12-11

**Authors:** Masahiro Tomita, Noriyuki Nagahara, Takaaki Ito

**Affiliations:** 1 Department of Pathology and Experimental Medicine, Kumamoto University Graduate School of Medical Sciences, 1-1-1 Honjo Chuo-ku, Kumamoto 860-8556, Japan; v2gd.sci.chem@gmail.com; 2 Isotope Research Center, Nippon Medical School; Tokyo 113-8602, Japan; noriyuki@nms.ac.jp

**Keywords:** mercaptopyruvate methyltransferase (MST), Western blotting, immunohistochemistry, mouse

## Abstract

3-Mercaptopyruvate sulfurtransferase (MST) is one of the principal enzymes for the production of hydrogen sulfide and polysulfides in mammalians, and emerging evidence supports the physiological significance of MST. As a fundamental study of the physiology and pathobiology of MST, it is necessary to establish the tissue distribution of MST in mice. In the present study, the expression of MST in various organs of adult and fetal mice was analyzed by Western blotting and enzyme-immunohistochemistry. Moreover, the histology of MST gene–deficient mice was examined. Western blotting revealed that all organs examined had MST. The brain, liver, kidneys testes, and endocrine organs contained large amounts of MST, but the lungs, spleen, thymus, and small intestine did not. Immunohistochemically, the MST expression pattern varies in a cell-specific manner. In the brain, neural and glial cells are positively stained; in the lung, bronchiolar cells are preferentially stained; in the liver, hepatocytes around central veins are more strongly stained; renal convoluted cells are strongly stained; and pancreatic islets are strongly stained. Fetal tissues were studied, and MST expression was found to be similar before and after birth. Histological observation revealed no remarkable findings in MST gene–deficient mice. The present study revealed fundamental information regarding the MST expression of various organs in adult and fetal mice, and the morphological phenotype of MST gene–deficient mice.

## 1. Introduction

Over the past two decades, hydrogen sulfide (H_2_S) has been revealed to play important roles in a variety of physiological processes, although H_2_S is widely known as a toxic gas. A number of studies have established that endogenous H_2_S acts as a gaseous signaling transducer in mammalian neurons and other cells, similar to nitric oxide and carbon monoxide [1,2]. H_2_S enhances the activity of *n*-methyl-d-aspartate receptors in neurons, leading to increased long-term potentiation in the hippocampus [1]. Interestingly, H_2_S made by non-sulfur bacteria was found to alleviate oxidative stress imposed by antibiotics, and to contribute to their resistance to antibiotics [3]. Additionally, other physiological roles of H_2_S, including mitochondrial redox signaling, cytoprotective effects, and neutrophil apoptosis, have also been reported in non-neuronal tissues [4,5,6,7,8,9,10].

Endogenous H_2_S is reported to be synthesized by four enzymes in mammalian tissues: cystathionine g-lyase (CSE, EC 4.4.1.1) [11], cystathionine β-synthase (CBS, EC 4.2.1.22) [12], mercaptopyruvate sulfurtransferase (MST, EC 2.8.1.2) [2,11,13,14], and thiosulfate sulfurtransferase (rhodanese; EC 2.8.1.1) [15]. Recently, more attention has been paid to MST, because it has been reported that MST is primarily responsible for H_2_S production in the central and peripheral nervous systems [16,17]. MST catalyzes the transsulfuration from mercaptopyruvate or thiosulfate to thiophilic acceptors and a persulfide is formed at the catalytic site cysteine as a reaction intermediate. The enzyme is well conserved among prokaryotes and eukaryotes, and has been implicated in important physiological roles related to sulfur metabolism. In rats, MST is expressed in various organs such as the brain, lungs, heart, kidneys, pancreas, and testes [18]. Immunohistochemical findings suggest the functional significance of MST in each organ, as the histological localization of MST is not diffuse, and cell-specific and spatial distributions were observed in the kidneys and liver [18]. It has been described that MST is also distributed in blood vessels [14]. Immunoelectron microscopic studies have also clarified the subcellular localization of MST in cytosol and mitochondria, suggesting MST as a defense against cyanide toxicity [19]. Physiological functions, including transsulfuration, H_2_S and polysulfides production [13], anti-oxidative stress and redox sensing [20,21], possible SO_x_ production [15], and anti-anxiety behavior [22], have also been reported.

In this study, as immunohistochemical investigation has not been performed in mice, we performed a detailed MST immunohistochemical investigation using Western blotting analyses. We describe histological observations of MST gene–deficient mice. Furthermore, we also studied changes in MST expression in the brain, lungs, and intestines during fetal development in order to clarify the functions of MST during this period and the involvement of MST in adaptation to an increasing oxygen concentration in the atmosphere.

## 2. Results

### 2.1. Western Blotting Analysis of MST in Mouse Organs

**Figure 1:**
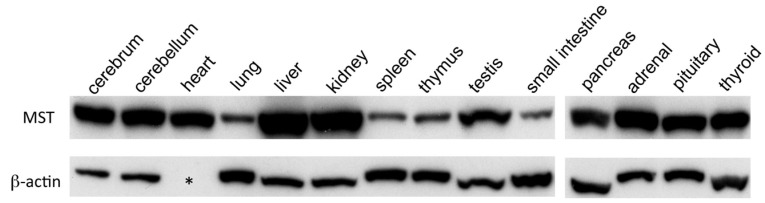
MST expression in various organs of mice by Western blotting. MST expression in the cerebrum, cerebellum, heart, lungs, liver, kidneys, spleen, thymus, testes, small intestines, pancreas, adrenal, pituitary, and thyroid glands; β-actin is not detected in the heart (asterisk).

The expression of MST was analyzed by Western blotting and was detected in all organs examined (Figure 1). Among them, the cerebrum, cerebellum, heart, liver, kidneys, testes, and endocrine organs, such as the pancreas, adrenal, pituitary, and thyroid glands, were rich in MST, but the amount of MST was relatively smaller in the lungs, spleen, thymus, and small intestines (Figure 1).

### 2.2. Immunohistochemical Distribution of MST in Various Organs

**Figure 2:**
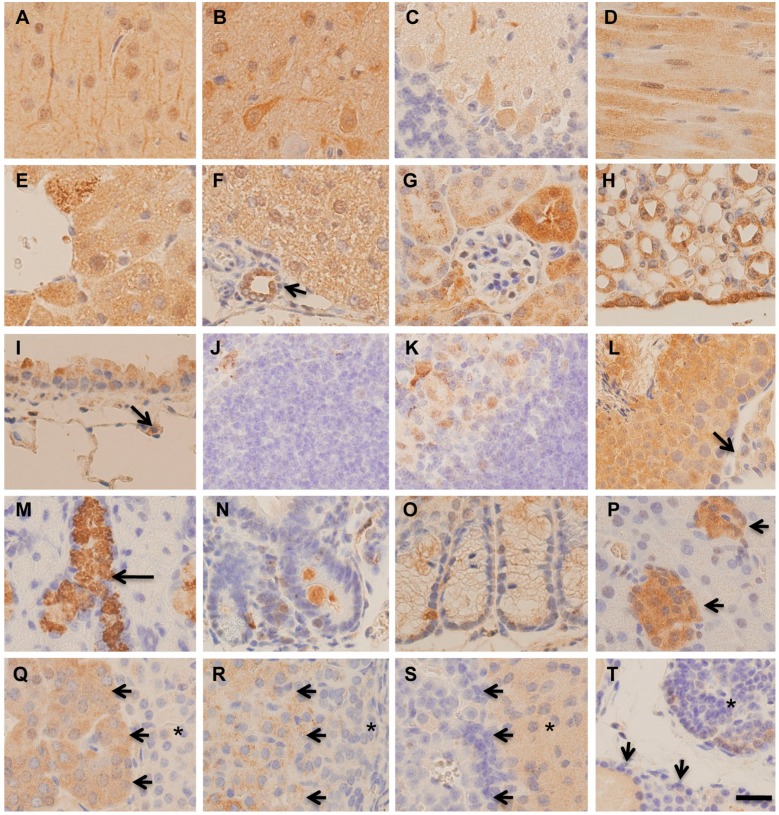
Immunohistochemistry for MST in various tissues of mice. (**A**) Cerebral cortex; (**B**) Thalamus; (**C**) Cerebellar cortex; (**D**) Heart muscle; (**E**) Central region of the liver; (**F**) Peri-portal liver tissue. Bile ductule (arrow); (**G**) Renal cortex; (**H**) Renal medulla and pelvic epithelium; (**I**) Bronchiole and alveolus. Type 2 alveolar cell (arrow); (**J**) Spleen; (**K**) Thymus; (**L**) Testis. Leydic cell (arrow); (**M**) Submandibular gland. Granular convoluted tubule (arrow); (**N**) Small intestines; (**O**) Colon; (**P**) Pancreas. Islet (arrows); (**Q**) Adrenal medulla (arrows). Reticular zone (asterisk); (**R**) Adrenal cortex. Fasciculata zone (arrows) and glomerular zone (asterisk); (**S**) Pituitary gland. Anterior lobe (arrows) and intermediate zone (asterisk); (**T**) Thyroid (arrows) and parathyroid (asterisk). Bar = 20 μm.

Immunohistochemistry for MST revealed that the staining intensity varied in a cell-specific and spatial manner. In the brain, both neural cells and glial cells were positively stained throughout the cerebrum (Figure 2A,B). In the cerebellum, the granular layer was negatively stained, some Purkinje cells were positively stained, and the molecular layer was positively stained (Figure 2C). Heart muscle cells showed positive staining diffusely in the cytoplasm (Figure 2D). In the liver, hepatocytes were positively stained, and the cells around the central veins were more strongly stained (Figure 2E,F). Bile ductular cells were positively stained (Figure 2F), and extrahepatic biliary epithelial cells were positively stained. In the renal cortex, glomeruli were negatively stained, while convoluted tubules were positively stained (Figure 2G). Proximal convoluted tubules were more strongly stained than distal ones (Figure 2G). In the renal medulla, collecting ducts were moderately stained, and renal pelvic epithelia were strongly stained (Figure 2H). In the lungs, the cytoplasm of bronchiolar Clara cells was moderately stained, but alveolar type 2 cells were weakly stained (Figure 2I). Alveolar macrophages were strongly stained. In the spleen, positive staining was rarely seen, except for in macrophages (Figure 2J), and in the thymus, epithelial cells in the medulla were positively stained (Figure 2K). In the testis, all cells in the seminiferous tubules were positively stained, and interstitial Leydic cells were weekly stained (Figure 2L). In the submandibular gland, granular convoluted tubule cells were strongly stained, while acinar glandular cells were negatively stained (Figure 2M). In the alimentary tract, esophageal squamous epithelia and gastric glandular mucosal epithelia showed weak staining. In the small intestine and colon, weak and occasional positive staining was observed, and a few strongly positive cells, presumably neuroendocrine cells, were scattered in the epithelia (Figure 2N,O). In the pancreas, islets were strongly stained, but acinar cells were negatively stained. Pancreatic ductal epithelia were positively stained. Adrenal medulla cells were positively stained (Figure 2Q). In the adrenal cortex, cells of the zona fasciculata were positively stained, although cells of the zona glomerulosa and zona reticularis were negative (Figure 2Q,R). In the pituitary gland, anterior lobe cells were rarely stained, and intermediate zone cells were moderately stained (Figure 2S). Parathyroid gland cells were positively stained, but thyroid follicular cells were negatively stained (Figure 2T).

### 2.3. Expression of MST in Fetal Mice Tissues

**Figure 3:**
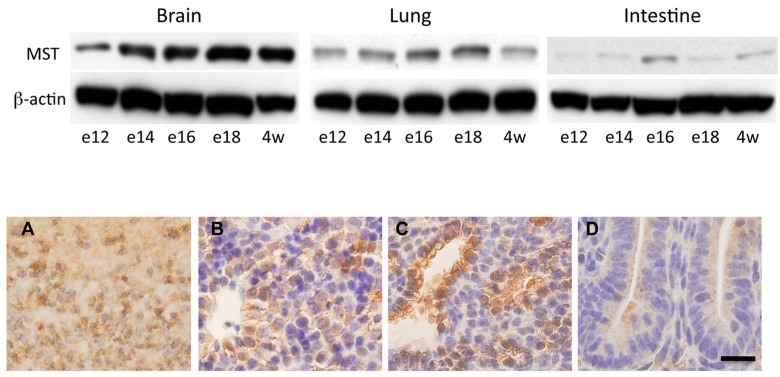
Expression of MST in fetal developing mice. (**Upper panel**) Western blotting analysis for MST in the developing fetal brain, lungs, intestines: e12 (fetal day 12), e14 (fetal day 14), e16 (fetal day 16), e18 (fetal day18), and 4w (4 week old); (**Lower panel**) Immunohistochemistry for MST in fetal day 16 mouse tissues; (**A**) Cerebral cortex; (**B**) Liver; (**C**) Lung; (**D**) Intestines. Bar = 20 μm.

The expression of MST in the developing fetal brain, lungs, and intestines was analyzed by Western blotting (Figure 3, upper panel). In the brain, the expression of MST was constantly detected throughout the observation period and did not change after birth. In the lungs and intestines, although the amount of MST was lower than that observed in the brain, MST was constantly expressed (Figure 3, upper panel). Immunohistochemically, fetal brain tissue cells were positively stained. Developing hepatocytes and bronchiolar cells were positively stained (Figure 3, lower panel). Fetal intestinal cells were weakly stained (Figure 3, lower panel).

### 2.4. Histological Changes in MST Gene–Deficient Mice

**Figure 4:**
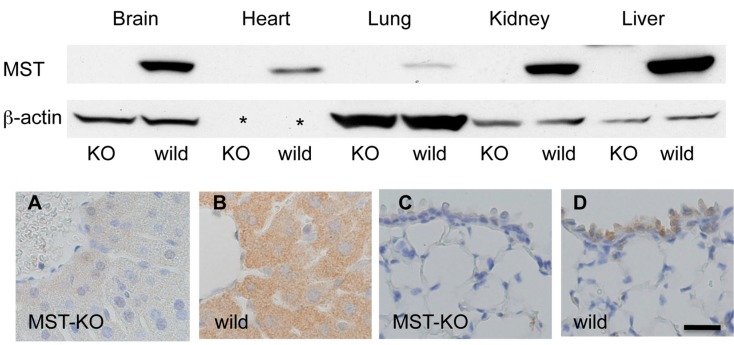
Expression of MST in MST gene–deficient mice. (**Upper panel**) No expression of MST in the brain, heart, lungs, kidneys, and liver of MST gene–deficient mouse (KO). β-actin is not detected in the heart (asterisk); (**Lower panel**) Immunohistochemistry for MST in an MST gene–deficient mouse (MST-KO) and a control mouse (wild); (**A**,**B**) Liver; (**C**,**D**) Lung. Bar = 20 μm.

Western blotting analysis showed that MST gene–deficient mice did not express MST (Figure 4, upper panel). However, a histological study does not clarify any morphological changes in the organs examined. Immunohistochemically, MST expression is reduced in MST gene–deficient mice in general (Figure 4, lower panel), but positive immunostaining is detected in some tissues of MST gene–deficient mice as the antibody for MST we used has a cross-reaction with rhodanese [18].

## 3. Discussion

MST is known as a multifunctional enzyme: it is a redox-controlling agent for H_2_S [15,22], polysulfide synthesis [2,13,14,15], anti-anxiety [22], and possible SO_x_ production [15]. In this study we confirmed that MST is ubiquitously expressed in various organs, and is localized in a cell-specific and spatial manner, which suggests special functions of MST in each organ.

MST serves as a thioredoxin-dependent redox-sensing molecular switch to change between inactivated and active states under oxidative conditions. This inactivation is performed via two pathways, with dimerization via a disulfide bond between MSTs, and formation of low-redox-potential sulfenic acid formed by a catalytic site cysteine. MST is reversibly reduced by thioredoxin. Inactivation of MST attenuates cysteine catabolism, which promotes the production of glutathione, glutaredoxin, and thioredoxin to defend against oxidative stress [15,21]. 

In this study, MST was found to be distributed throughout the mouse brain, and shows positive staining in both neural and glial cells, although our previous study in the rat brain showed preferential glial staining [22], and Wrobel et al. have proposed that it is controversial whether MST is localized in neurons or glial cells in the brain [23]. The different results in our studies may be due to the immunostaining methods applied: fluorescent immunohistochemistry for rat formalin-fixed frozen sections versus enzyme immunohistochemistry for mouse formalin-fixed paraffin sections. Zhao et al. [24] also reported that MST activities are detected in many parts of the brain, and there appear to be no significant differential distributions; however, they reported MST to be expressed in glial but not neural cells [24]. Other studies have reported positive staining in neurons, but not in glial cells [12]. As to the function of MST, Kimura et al. [25] proposed that MST might be an important enzyme for the synthesis of H_2_S in the brain. H_2_S in the central nervous systems is proposed to be involved in physiological processes such as the enhancement of hippocampal long-term potentiation. Li et al. studied chronic hypoxia in rat brains and discovered that hypoxia could activate MST expression and MST-mediated H_2_S production [26]. In contrast, acute strokes appear to decrease MST expression, and MST is significantly down-regulated after a permanent middle cerebral artery occlusion [24]. 

In tissues other than neural tissues, MST was detected in various organs, such as the pituitary, thymus, thyroid, adrenal, and submandibular glands, lungs, kidneys, liver, heart, spleen, pancreas, intestines, and testes in mice. The present study supports our previous observations of the rat kidneys and liver, in which we found cell-specific expression of MST in the kidneys and region-specific graduation in the liver. In addition, it is noteworthy to mention that MST is highly expressed in neuroendocrine organs: pancreas (pancreatic islets compared with pancreatic acinar cells), pituitary glands, adrenal glands, and parathyroids. A very few reports, however, have reported the relationship between MST and neuroendocrine tissues. For instance, Czabo et al. reported that a hyperglycemic state reduces the activity of vascular MST, causing suppressed vasorelxation via H_2_S [27]. H_2_S is reported to control insulin secretion in pancreatic β cells [9], although endogenous H_2_S in pancreatic β cells has been estimated to be biosynthesized mainly via CSE and CBS pathways. Considering its high expression in pancreatic islets, MST also might be involved in H_2_S production. The association between MST and neuroendocrine systems remains to be clarified in detail.

Our immunohistochemical study in mice during developmental stages showed that MST exhibits high expression in organs during this period, and that it is similarly expressed in adult organs. Further study is needed to fully understand the relationship between MST expression and development [28]. In addition to the significance of MST in fetal organs, the fact that no obvious morphological abnormalities were seen in MST gene–deficient mice might indicate the difficulty of analyzing the physiological significance of this enzyme.

## 4. Materials and Methods

### 4.1. Animals

For Western blotting and immunohistochemical studies of adult mice, six-week old C57/BL mice were used (Japan SCL, Shizuoka, Japan). For Western blotting and immunohistochemical studies of fetal mice, Institute of Cancer Research (ICR) mice were purchased from Japan SLC (Shizuokam, Japan). Mice were mated overnight, and the day of the discovery of the vaginal plug was counted as fetal day 0. Four-week-old ICR male mice were used for comparison with fetal mice. MST gene–deficient mice were produced according to a previously described method [22] (Unitech, Chiba, Japan). All animal experiments were conducted in accordance with the guidelines of the Animal Care and Use Committee of Kumamoto University.

### 4.2. Western Blotting

For Western blotting analysis of C57/BL adult mice, six animals were used in total. The tissues from the cerebrum, cerebellum, heart, lungs, kidneys, spleen, thymus, testes, small intestines, and pancreas were sampled from three animals, and frozen until the time of analysis. The tissues from adrenal, pituitary and thyroid glands, including the parathyroid, were gathered from six animals. Thyroid tissues were acquired under a dissecting microscope. For Western blotting analysis of fetal ICR mice, the tissues from the brain, lungs, and intestines were acquired from 14 fetuses from a gestational day 12 mouse, eight fetuses from a gestational day 14 mouse, five fetuses from a gestational day 16 mouse, three fetuses from a gestational day 18 mouse, and three 4-week-old male mice. In addition, the brain, heart, lungs, kidneys, and liver were obtained from three 6-week old male MST gene–deficient mice and three 6-week old male wild0type mice for Western blotting analysis. 

The tissues were homogenized on ice with ice-cold homogenizing buffer (5 mM Tris, 0.25 M sucrose, 2 mM EDTA, 2 mM ethylene glycol bis(2-aminoethyl ether)-tetraacetic acid (EGTA)) containing the protease inhibitors phenylmethane sulfonyl fluoride (PMSF, 0.5 mM), dithiothreitol (DTT, 0.5 mM), and leupeptin (5 mg/mL), clarified by centrifugation, and resuspended in lysate buffer (50 mM Tris, 150 mM NaCl, 25 mM NaF, 25 mM glycerophosphate, 2 mM EDTA, 2 mM EGTA, 0.3% NP-40, 0.5 mM PMSF, 0.5 mM DTT, 5 mg/mL leupeptin). The lysate was centrifuged and the concentration of the supernatant protein extract was determined using the Bio-Rad protein assay kit (Bio-Rad Laboratories, Irvine, CA, USA). Aliquots of 30 μg of protein were subjected to SDS-PAGE (in 8%–12% gels), and the protein was then transferred to nitrocellulose membranes. The blots were blocked with 5% non-fat milk in phosphate-buffered saline solution. Rabbit antibody against rat MST [18] was used for Western blotting analysis. The filters were incubated with the primary antibody, and then with horseradish peroxidase-conjugated secondary antibody, and the stained proteins were visualized with an enhanced chemiluminescence detection kit (Amersham Pharmacia Biotech, Uppsala, Sweden). β-actin was also detected in each sample.

### 4.3. Histology and Immunohistochemistry

The brain, heart, lungs, kidneys, spleen, thymus, testes, submandibular gland, esophagus, stomach, small intestines, colon, and pancreas tissues from three 6-week-old C57/BL male mice, the adrenal, pituitary, and thyroid gland tissues from six 6-week-old C57/BLmale mice, the fetal tissues from six fetuses from a gestational day 16 ICR mouse, the brain, heart, lungs, kidneys, liver, and small intestines tissues from three 4-week-old male ICR mice, and the brain, heart, lungs, kidneys, liver, pancreas, testes, adrenal, pituitary, and thyroid gland tissues from six MST gene-deficient male mice were fixed in phosphate-buffered 4% paraformaldehyde for two days, and embedded in paraffin. The paraffin-embedded sections of all the embedded samples were rehydrated with xylene and graded ethanol solutions, and stained with hematoxylin and eosin stain. For immunohistochemistry, after treatment with 0.3% hydrogen peroxide, the sections were heated at 97 °C for 15 min in antigen retrieval solution (pH6 citrarate buffer solution). After treatment with Protein Block Serum-Free Reagent (Dako, Glostrup, Denmark) for 20 min, the sections were treated with rabbit anti-rat MST antibody overnight at 4 °C. After washing, the sections were treated with anti-rabbit IgG conjugated with HRP-polymer (Dako) for 60 min at room temperature. The sections were further treated with diaminobenzidine-hydrogen peroxide solution, and counterstained with hematoxylin. 

## Abbreviations

The following abbreviations are used in this manuscript:

CSE	cystathionine g-lyase
CBS	cystathionine b-synthase
H_2_S	Hydrogen sulfide
MST	3-mercaptopyruvate sulfurtransferase
